# Transcranial Direct Current Stimulation over the Dorsolateral Prefrontal Cortex in Schizophrenia: A Quantitative Review of Cognitive Outcomes

**DOI:** 10.3389/fnhum.2017.00044

**Published:** 2017-02-02

**Authors:** Joshua E. Mervis, Riley J. Capizzi, Elias Boroda, Angus W. MacDonald

**Affiliations:** ^1^Department of Psychology, University of MinnesotaMinneapolis, MN, USA; ^2^Department of Neuroscience, University of MinnesotaMinneapolis, MN, USA; ^3^Department of Psychiatry, University of Minnesota Medical SchoolMinneapolis, MN, USA

**Keywords:** cognition, dorsolateral prefrontal cortex, quantitative review, Schizophrenia, transcranial direct current stimulation

## Abstract

Cognitive deficits are a core and disabling feature of psychotic disorders, specifically schizophrenia. Current treatments for impaired cognition in schizophrenia remain insufficient. Recent research suggests transcranial direct current stimulation (tDCS) targeting the dorsolateral prefrontal cortex can potentiate cognitive improvements in healthy individuals and those with psychiatric conditions, such as schizophrenia. However, this burgeoning literature has not been quantitatively evaluated. Through a literature search and quantitative review, we identified 194 papers on tDCS, psychosis, and cognition. Selection criteria included pre/post design and sham control to achieve specific sham-adjusted effect sizes. The 6 retained studies all address schizophrenia populations and include single and repeated stimulation, as well as within and between subject designs. Small positive effects were found for anodal stimulation on behavioral measures of attention and working memory, with tentative findings for cognitive ability and memory. Cathodal stimulation yielded a small positive effect on behaviorally measured cognitive ability. Neurophysiological measures of attention showed a small to medium down-modulation effect for anodal stimulation. Implications of these findings and guidelines for future research are discussed. As revealed by this report, due to the paucity of data available, much remains unknown regarding the clinical efficacy of tDCS in schizophrenia.

## Introduction

Impaired cognition is a significant and disabling feature of psychotic disorders such as schizophrenia. Deficits in executive functions (working memory, attention, response inhibition) are the most commonly reported, and the most predictive of functional outcome (Green, 1996). Despite the central role of these impairments, current treatments, including pharmacological interventions, have proven ineffective at ameliorating cognitive dysfunction (Fusar-Poli et al., 2015). New or adjunctive treatment options are needed.

A large body of evidence implicates impaired frontal cortical activity as a causal factor in cognitive dysfunction in schizophrenia (Minzenberg et al., 2009). Specifically, hypoactivation of the dorsolateral prefrontal cortex (DLPFC) has been suggested as the core deficit (Potkin et al., 2009; Lesh et al., 2011). Given the pivotal role of the DLPFC in mediating a wide range of executive functions (Niendam et al., 2012), interventions that target this region are of great clinical interest. Non-invasive methods of neuromodulation provide a safe, cost-effective and robust means to enhance DLPFC function.

Transcranial current stimulation (tCS) is a non-invasive neuromodulation technique that uses small, specifically directed electrical currents to alter cortical brain activity (Nitsche and Paulus, 2001). Though there are several forms of tCS, transcranial direct current stimulation (tDCS) has by far been the most commonly employed. TDCS involves the use of two electrodes, a positively charged anode and a negatively charged cathode. Studies in both animals and humans demonstrate that anodal stimulation produces a shift in excitability that depolarizes neurons, while cathodal stimulation has opposite effects (Nitsche and Paulus, 2000; Cambiaghi et al., 2010). Though the mechanisms underlying tDCS are still under investigation, it is postulated that these shifts in excitability are induced by altering membrane polarization at the cellular level (Fritsch et al., 2010; Kronberg et al., 2017). Due to its safety (Bikson et al., 2016), tolerability, and low cost, the use of tDCS has grown substantially. Recent research demonstrates that tDCS targeted to the DLPFC has the ability to potentiate changes in cognition in both healthy individuals (Fregni et al., 2005) and those with various psychiatric conditions, such as schizophrenia (Dedoncker et al., 2016).

As a clinical intervention, the use of tDCS to enhance cognition in schizophrenia is especially promising. Anodal tDCS, directed at the DLPFC, has now been evaluated in several trials as a possible rehabilitation technique or adjunct to existing treatments (Minzenberg and Carter, 2012; Palm et al., 2016). However, research has indicated contradictory effects of stimulation in some patient populations (Berryhill et al., 2014) and differential effects on various cognitive domains are not well understood.

To address these ambiguities, we undertook a quantitative review of studies on tDCS in schizophrenia using the PubMed database and identified 194 articles. This number was reduced to 6 articles after excluding studies on populations without psychosis, without cognitive outcomes, and including only those with a sham stimulation condition to create a sham-adjusted effect size. Study outcomes and heterogeneity of designs were aggregated and variance-weighted.

## Methods

### Literature search

A literature search was conducted in the PubMed Database searching titles and abstracts for the following key words and Boolean terms: (“psychosis” OR “schizophrenia” OR “schizoaffective disorder” OR “bipolar disorder”) AND (“tDCS” OR “direct current”). Published articles were collected up until May 2016 returning 194 results.

### Eligibility criteria

**Figure 1:**
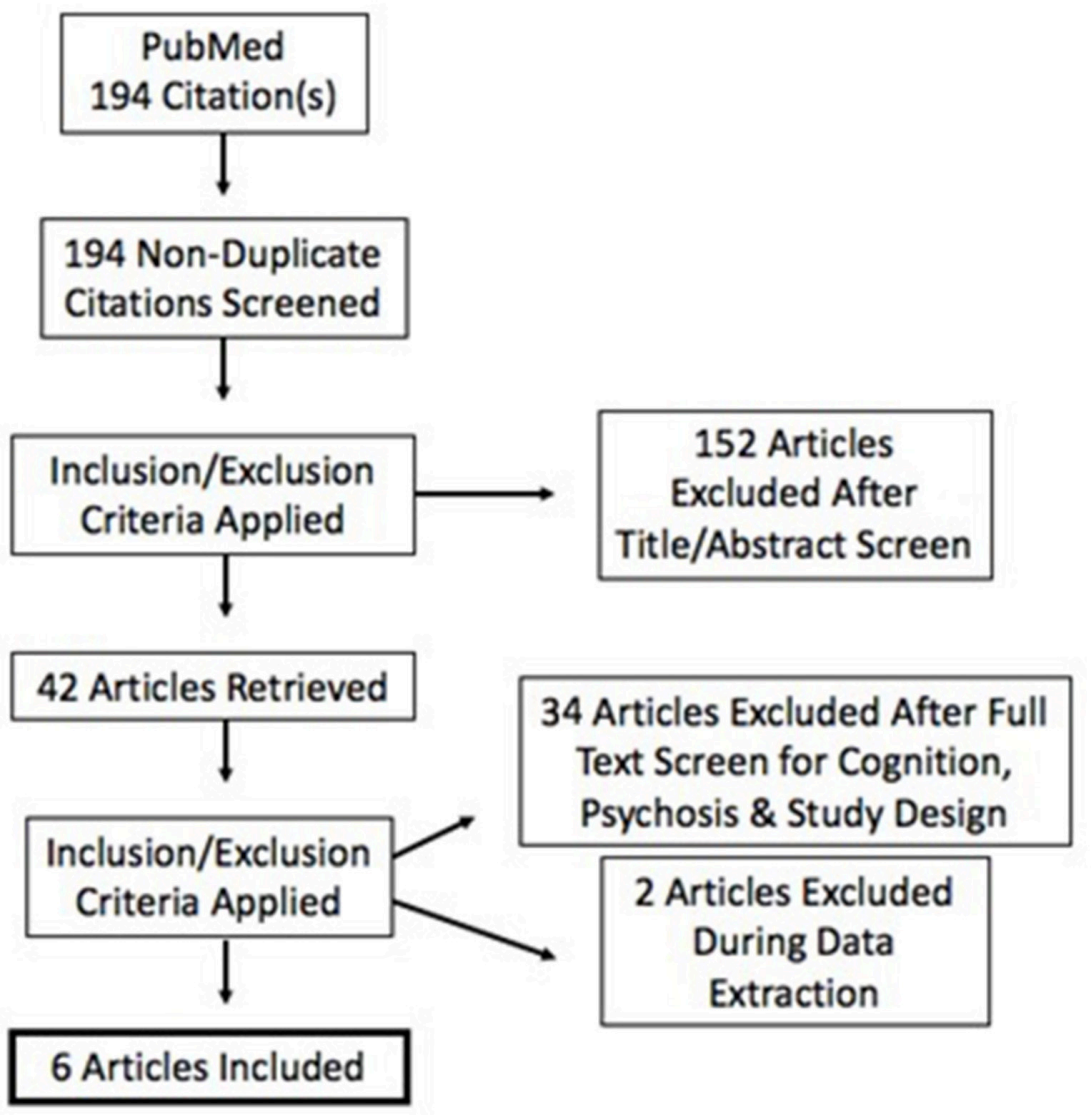
**Literature Search and Study Eligibility Determination**.

Criteria for inclusion were: (a) psychosis; (b) randomized and sham-controlled designs; (c) pre-post within-subject or between-subject designs. Duplicates, reviews, case studies, and studies with <3 participants were excluded. Studies were also excluded due to the inability to calculate independent groups pre-post effect sizes (Becker, 1988; Lipsey and Wilson, 1993) See Figure 1. Specific study characteristics extracted for discussion are presented in Table 1. No formal quality assessment was performed.

**Table 1:** **Anodal tDCS studies: cognitive domains, measures, and design parameters**.

**Study**	**Measures**	**Active electrode**	**Reference electrode**	**Stimulation target**	**On/Offline**	**Amplitude (mA)**	**Sessions**	**Unweighted effect size (d_uw_)**	**Weighted effect size (d_w_)**	**d_w_ Ranking (high/low)**
**ATTENTION**										
Dunn et al., 2016	P300 (μV)	Fp1/Fp2	Upper Arm (R)	Bilateral DLPFC	Offline	1	2	0.16	0.78	8
Dunn et al., 2016	Mismatch Negativity (μV)	Fp1/Fp2	Upper Arm (R)	Bilateral DLPFC	Offline	1	2	−1.54	−4.05	22
Mean Effect	(Neurophysiological)							−0.69	−0.44	
Smith et al., 2015	MCCB Attention-Vigilance (accuracy)	F3	Supraorbital (R)	Left DLPFC	Offline	2	5	0.49	3.01	2
Smith et al., 2015	MCCB Reasoning/Problem Solving (seconds)	F3	Supraorbital (R)	Left DLPFC	Offline	2	5	0.32	1.99	4
Mean Effect	(Behavioral)							0.40	0.40	
**MEMORY**										
Smith et al., 2015	MCCB Verbal Learning (recall and recognition scores)	F3	Supraorbital (R)	Left DLPFC	Offline	2	5	0.21	1.33	7
Smith et al., 2015	MCCB Visual Learning (recall and recognition scores)	F3	Supraorbital (R)	Left DLPFC	Offline	2	5	0.10	0.55	10
Mean Effect								0.21	0.16	
**PROCESSING SPEED**										
Smith et al., 2015	MCCB Speed of Processing (accuracy)	F3	Supraorbital (R)	Left DLPFC	Offline	2	5	−0.18	−1.12	20
Palm et al., 2016	Trail-Making Test A (seconds)	F3	Supraorbital (R)	Left DLPFC	Offline	2	10	−0.14	−0.49	17
Palm et al., 2016	Trail-Making Test B (seconds)	F3	Supraorbital (R)	Left DLPFC	Offline	2	10	0.11	0.41	11
Mean Effect								−0.16	−0.16	
**SOCIAL COGNITION**										
Rassovsky et al., 2015	TASIT (total score)	Fp1/Fp2	Upper Arm (R)	Bilateral DLPFC	Offline	1	1	−0.12	−0.59	18
Rassovsky et al., 2015	PONS (total score)	Fp1/Fp2	Upper Arm (R)	Bilateral DLPFC	Offline	1	1	−0.13	−0.65	19
Rassovsky et al., 2015	FEIT (accuracy)	Fp1/Fp2	Upper Arm (R)	Bilateral DLPFC	Offline	1	1	0.44	2.02	3
Rassovsky et al., 2015	MSCEIT (total score)	Fp1/Fp2	Upper Arm (R)	Bilateral DLPFC	Offline	1	1	0.02	0.08	16
Smith et al., 2015	MCCB Social Cognition (total score)	F3	Supraorbital (R)	Left DLPFC	Offline	2	5	0.26	1.54	6
Mean Effect								0.14	0.15	
**WORKING MEMORY**										
Hoy et al., 2016^*^	2-back (Letters) (accuracy)	F3	Supraorbital (R)	Left DLPFC	Offline^***^	2	1–2	−0.46	−1.18	21
Nienow et al., 2016^**^	2-back (Words) (accuracy)	F3	Supraorbital (R)	Left DLPFC	Online	1	28	0.53	0.24	14
Nienow et al., 2016^**^	2-back (Pictures) (accuracy)	F3	Supraorbital (R)	Left DLPFC	Online	1	28	0.58	0.31	13
Palm et al., 2016	Self-Ordered Pointing Task (accuracy)	F3	Supraorbital (R)	Left DLPFC	Offline	2	10	0.19	0.73	9
Smith et al., 2015	MCCB Working Memory (recall score)	F3	Supraorbital (R)	Left DLPFC	Offline	2	5	0.51	3.12	1
Mean Effect								0.21	0.23	
**COGNITIVE ABILITY**										
Nienow et al., 2016^**^	MCCB Composite (total score)	F3	Supraorbital (R)	Left DLPFC	Online	1	28	0.14	0.12	15
Rassovsky et al., 2015	MCCB Composite (total score)	Fp1/Fp2	Upper Arm (R)	Bilateral DLPFC	Offline	1	1	0.07	0.34	12
Smith et al., 2015	MCCB Composite (total score)	F3	Supraorbital (R)	Left DLPFC	Offline	2	5	0.30	1.89	5
Mean Effect								0.17	0.20	

*All studies stimulated for 20 min at a time, electrode area was 35 cm^2^ for all but Smith et al. (2015) which was 5.08 cm^2^*.

* *Randomized between sessions for transcranial alternating, direct, and sham current stimulation. Only tDCS and sham conditions included in review*.

** *Includes cognitive remediation training (48 h) for both tDCS and sham groups*.

*** *Study includes stimulation concurrent to task, although effect sizes were calculated from pre-post assessments*.

### Quantitative review

#### Effect sizes

Effect sizes were calculated by combining elements of repeated measures and independent groups designs as described by previous meta-analytic work (Becker, 1988; Morris and DeShon, 2002, Equation 6):

$$\begin{array}{l} d=\frac{Stimulation\;Mean_{post}-\;Stimulation\;Mean_{baseline}}{Stimulation\;SD_{baseline}}\\ \;-\;\frac{Sham\;Mean_{post}-\;Sham\;Mean_{baseline}}{Sham\;SD_{baseline}} \end{array}$$

Baseline standard deviations are assumed to be more comparable across studies before different treatments are applied. Sham-adjustment is important because research has observed non-zero changes in control groups (sham) over time (Lipsey and Wilson, 1993; Carlson and Schmidt, 1999).

#### Sampling variance

Sampling variance calculations were selected to match the combined effect size (Becker, 1988), drawn from Morris and DeShon (2002), computing each group's variance separately (Equation A1) and adding them together, where *df* = n−1, *d* is the effect size, and *c* is the bias function $1-\frac{3}{4df-1}$ (Equation 23):

$$\begin{array}{l} sampling\;variance\;=\left(\frac{1}{n}\right)\left(\frac{df}{df-2}\right)\left(1\;+\;n*d^{2}\right)-\frac{d^{2}}{{c\left(df\right)}^{2}} \end{array}$$

Meta-analytic procedures detailed in Lipsey and Wilson (2001) and Morris and DeShon (2002) were used to calculate weights as the inverse of the squared standard error for each effect size. Variance-weighted mean effect sizes (d_W_) and mean effect sizes without weights (d_UW_) should be interpreted carefully as not all studies examined each cognitive domain discussed. There are an inadequate number of measures in each domain to detect a significant effect for a specific hypothesis (e.g., *Z*-test), even if sample-dependent measures are treated as sample-independent.

## Effect sizes for cognition

Sham-adjusted effect sizes for anodal stimulation are reported in Table 1. As a complement to effect sizes, ranks for variance-weighted effect sizes are also included in the table, with larger, positive descriptive effects ranked highest and the rest in descending order. Confidence intervals are included below as a measure of variability. For comparison to the greater literature (without any specific hypothesis testing) effect sizes are discussed according to Cohen's conventions of 0.2, 0.5, and 0.8 as putative measures for small, medium, and large descriptive, non-inferential effects (Cohen, 1992). These distinctions were originally a proposed route for accurate foresight in power analysis and are not strictly indicative of clinical efficacy (Abelson, 1985; Prentice and Miller, 1992). Results from other areas provide a benchmark: small classes rather than large had an effect of 0.20 on educational achievement (Hedges and Stock, 1983), therapy for test anxiety in college students showed an effect of 0.58 on anxiety and test performance (Harris, 1988). Other effects beneath Cohen's conventions that may be worth implementing include individualized education program's effects on achievement at 0.10 (Bangert-Drowns et al., 1983) and 0.17 (Hood, 1991). Therefore, these conventions should not underrepresent the importance of the effects of stimulation.

### Overview

The 194 studies were screened according to inclusion and exclusion criteria at title, abstract, and full text levels and subsequently reduced to 6 studies for quantitative review. A flow diagram indicating successive exclusion is provided in Figure 1. Although, search terms were determined in order to garner citations involving psychosis, it is important to note that our search rendered only populations diagnosed with schizophrenia and schizoaffective disorder. All studies evaluated individuals with schizophrenia; three included schizoaffective disorder. All articles meeting eligibility criteria stimulated the DLPFC. Cathodal stimulation is reported where applicable or omitted from results when not. Domains of cognition included in the retained articles are discussed herein.

### Attention

Smith et al. (2015) reported the only behavioral measures of attention, with a variant of the continuous performance task. The mean effect size for anodal stimulation was small to medium (d_w_ = 0.40, 95% CI: −0.15, 0.96; d_uw_ = 0.40). No behavioral measures were used with cathodal stimulation in the studies reviewed. Only Dunn et al. (2016) included neurophysiological measures of attention and error, using an auditory oddball task to elicit event related potentials, specifically P300 and mismatch negativity. Different effects were observed between the anodal stimulation group (d_w_ = −0.44, 95% CI: −1.17, 0.28; d_uw_ = −0.69) and the cathodal stimulation group (d_w_ = 0.10, 95% CI: −.53, 0.73; d_uw_ = 0.10). These neurophysiological outcomes are included for the purposes of the review, yet are not a part of other calculated mean effects.

### Memory

Only Smith et al. (2015) included measures for memory with a letter-number span task. The mean anodal effect was marginal to small (d_w_ = 0.16, 95% CI: −.41, 0.73; d_uw_ = 0.21).

### Processing speed

Two studies employed symbol-coding tasks to measure processing speed. The mean anodal effect for processing speed was marginal (d_w_ = −0.16, 95% CI: −.78, 0.46; d_uw_ = −0.16).

### Social cognition

The mean anodal effect for social cognition was marginal (d_w_ = 0.15, 95% CI: −0.44, 0.75; d_uw_ = 0.14) as calculated from two studies that included the same broad measure (Mayer-Salovey-Caruso Emotional Intelligence Test). For cathodal conditions, the effect (d_w_ = 0.06, 95% CI: −0.39, 0.51; d_uw_ = 0.06) was calculated as the mean effect for all measures included in the one study that examined social cognition (Rassovsky et al., 2015; see Table 1).

### Working memory

The mean anodal effect for working memory was small (d_w_ = 0.23, 95% CI: −0.31, 0.77; d_uw_ = 0.21). The effect was calculated from four studies using n-back tasks to measure working memory (variants of the N-Back task). Two of these studies carried out anodal tDCS concurrent with administration of the working memory task (Hoy et al., 2016; Nienow et al., 2016). Nienow et al. (2016) used picture and word n-backs to avoid direct practice effects from treatment sessions. The combined interpretation suggests a small effect at this time. The variance-weighted mean effect without Nienow et al. and Hoy et al. is almost double, but still a small effect (d_w_ = 0.39, 95% CI: −0.23, 1.01; d_uw_ = 0.35). These three studies were particularly low in variability, which also explains the difference between weighted and unweighted effect sizes when Nienow et al. is excluded.

### Cognitive ability

Across three studies, the mean anodal effect for general cognitive ability was small to marginal (d_w_ = 0.20, 95% CI: −0.37, 0.76; d_uw_ = 0.17). Although, one of the studies included here also employed cognitive remediation in tandem with tDCS (Nienow et al., 2016), this summary category also has the advantage of using the same measure (Matrics Consensus Cognitive Battery; Nuechterlein and Green, 2006). Only Rassovsky et al. (2015) included a cathodal stimulation condition, for which the effect size was small (d_w_ = 0.29, 95% CI: −0.61, 1.18; d_uw_ = 0.29). For the studies without a direct measure for general cognitive ability (Hoy et al., 2016; Palm et al., 2016), effect sizes within the studies were averaged as a general measure. Including the additional studies, general cognitive ability showed a marginal mean weighted effect for anodal stimulation (d_w_ = 0.06, 95% CI: −0.41, 0.52; d_uw_ = 0.06).

## Effect sizes for studies by methods used

Unless otherwise noted, methodological issues are discussed across all domains and are not specific. Examining bilateral stimulation through the two studies that used such a montage (Rassovsky et al., 2015; Dunn et al., 2016) resulted in different effects for behavioral and neurophysiological outcomes. For the purposes of this review, bilateral stimulation refers to montages containing two stimulating electrodes of the same polarity with a separate, third electrode serving as the reference electrode. Therefore, they were not averaged so as to not understate their differences, nor were confidence intervals reported. These effects were positive and small to marginal for mean behavioral outcomes in Rassovsky et al. (2015; d_w_ = 0.24, d_uw_ = 0.05) with anodal stimulation and with cathodal stimulation medium to marginal (d_w_ = 0.51, d_uw_ = 0.11). For neurophysiological outcomes (event related potential measures of P300 and mismatch negativity), the mean effects of anodal stimulation were negative and medium to large (d_w_ = −1.64; d_uw_ = −0.69), whereas cathodal stimulation resulted in a marginal to near-medium effect (d_w_ = 0.48, d_uw_ = 0.10; Dunn et al., 2016). These divergent effects likely result from different results within the study, particularly given a large negative effect for one measure as seen in Table 1. Unilateral anodal stimulation (d_w_ = 0.06, 95% CI: −0.48, 61; d_uw_ = 0.23) was used by four studies (Smith et al., 2015; Hoy et al., 2016; Nienow et al., 2016; Palm et al., 2016) with behavioral outcomes and yielded marginal to small effect sizes calculated using the average of all effects in each behavioral study. Only one study (Hoy et al., 2016) showed a negative finding in this area, but that study only included a single measure (working memory).

Current intensity was also examined for differences in effects between 1 and 2 mA stimulation on behavioral outcomes and was only conducted for anodal stimulation. Based on two studies (Rassovsky et al., 2015; Nienow et al., 2016), marginal to small effects were found for 1 mA stimulation (d_w_ = 0.09, 95% CI: −0.76, 0.93; d_uw_ = 0.24). Based on three studies (Smith et al., 2015; Hoy et al., 2016; Palm et al., 2016), marginal effects were observed for 2 mA stimulation (d_w_ = 0.04, 95% CI: −0.51, 0.60; d_uw_ = −0.05). Only one study, which was excluded for only using a post-test, did have multiple amplitudes (Hoy et al., 2014) and only found improvements for the 2 mA anodal stimulation.

The number of active stimulation sessions constitutes another methodological difference common in the literature. Two studies (Rassovsky et al., 2015; Hoy et al., 2016) using a single session of anodal tDCS found marginal to small negative effects (d_w_ = −0.13, 95% CI: −0.85, 0.59; d_uw_ = −0.20) that appear driven by one study, highlighting the challenge of summarizing the literature at this time. Three studies (Smith et al., 2015; Nienow et al., 2016; Palm et al., 2016) using more than one session of anodal tDCS, ranging from 5 to 28 sessions showed a positive marginal to small effect (d_w_ = 0.19, 95% CI: −0.42, 0.80; d_uw_ = 0.24). Currently, a direct trend has not been identified between the amount of stimulation sessions and cognitive enhancement. Such empirical evidence would prompt establishing an accepted dose of stimulation.

The most studied areas of cognition with tDCS in psychosis are working memory, attention, and cognitive ability (Smith et al., 2015; Hoy et al., 2016; Nienow et al., 2016; Palm et al., 2016). This may be due to their overall importance in the literature for the treatment of cognitive deficits in psychosis. Variety exists in measurement time points used in study designs, for example, post-test only (Hoy et al., 2014, 2015), or stimulation concurrent to task and assessment (Vercammen et al., 2011; Schretlen et al., 2014; Reinhart et al., 2015a,b). This is an especially pertinent source of variability as recent research has demonstrated that tDCS effects are highly state dependent (Elmasry et al., 2015; Gill et al., 2015; Dedoncker et al., 2016), suggesting that the combination of a task with stimulation might yield greater modulation of cognitive domains. As an example, one of our reviewed studies (Nienow et al., 2016) used stimulation concurrent with cognitive remediation and found positive effects. Another important source of variability may stem from differences in the overall electrode montage. It has been shown that even minor changes in placement of the reference electrode affect the distribution and intensity of electrical current (Bai et al., 2014).

## Summary

This report captures the current state of the literature as it pertains to the cognitive outcomes from tDCS targeted at the DLPFC in schizophrenia. Although, none of the effects observed in this small sample rule-out the possibility of null effects, we were able to quantitatively summarize current knowledge and provide the central tendency of the effects on cognitive outcomes following tDCS over the DLPFC. Small effects of anodal stimulation were observed on behavioral measures of attention and working memory. More tentative small effects were observed for cognitive ability and memory, with marginal effects observed on processing speed. Cathodal stimulation paired with behavioral outcomes suggested a small effect on cognitive ability and a marginal one for social cognition, though this area of the literature is currently underdeveloped.

Neurophysiological measures were restricted to attention and were associated with a small to medium negative effect for anodal stimulation that is driven by a strong modulation of mismatch negativity (Dunn et al., 2016). A marginal effect for cathodal stimulation was also found. A closer reading of that study suggests minor differences in negative symptoms at baseline in the anodal stimulation group. However, another study not indexed in the PubMed database showed a null finding for several neurophysiological measures (Knechtel et al., 2014) included in Dunn et al. (2016).

Bilateral stimulation with behavioral measures seems to produce tentative small effects with anodal stimulation and medium effects with cathodal. Behavioral measures with a unilateral montage were only assessed with anodal stimulation, which appears to produce either marginal or small effects. More research in unilateral stimulation is important, even though it is more commonly used than bilateral stimulation. Future reviews might seek to address the more specific placement of active or reference electrodes according to the international 10–20 system or more detailed schemas where available.

For behavioral outcomes, no particular current intensity seemed critical for modulation with anodal stimulation. Some studies incorporate an alternating current condition and find promising effects (Göder et al., 2013; Hoy et al., 2016). The number of anodal stimulation sessions differed such that a single session of stimulation showed a marginal to small negative effect, whereas multiple sessions showed a marginal to small positive effect.

One general limitation of this review is that the overall and domain-specific weighted averages for cognition must be interpreted carefully, as the sample size and statistical dependence of measures makes inference premature, and few studies report power analyses. Researchers must report means and standard deviations for all groups and time points or other statistics to aid in producing effect sizes. Additionally, few authors studying cognition with multiple measurement time points use neurophysiological measures. One of the largest discrepancies in this review emerges from that fact. With anodal stimulation, behavioral measures of attention showed small, non-significant improvements, but neurophysiological measures showed a decline with a near-medium effect size. More studies using neurophysiologically grounded outcomes (i.e., EEG, fMRI) are critical to understanding the efficacy of tDCS as a potential modulator for cognition in schizophrenia.

This review of tDCS over the DLPFC in schizophrenia highlights methodological heterogeneity that reflects no current gold standard. Although, the review was conducted without specific hypothesis testing, a positive effect is observed for anodal stimulation on several domains of behaviorally measured cognition, with a negative effect on neurophysiologically measured attention. Some support exists for a positive effect of cathodal stimulation on cognition with measures that are behavioral. Future research with larger sample sizes and combined behavioral and neurophysiological outcomes in the same studies are needed to push the field forward.

## Author contributions

JM co-designed the project, conducted the analysis, and wrote all drafts of the manuscript. RC co-designed the project, supervised data collection, assisted in the analysis, and co-wrote manuscript drafts. EB provided conceptual guidance on project design and co-wrote all drafts of the manuscript. AM designed the project, supervised data analysis, and co-wrote all drafts of the manuscript.

## Funding

This project was funded by a Graduate Summer Research Fellowship from the Department of Psychology at the University of Minnesota and a Sambol Family Foundation Grant to JM.

### Conflict of interest statement

The authors declare that the research was conducted in the absence of any commercial or financial relationships that could be construed as a potential conflict of interest.
